# Considerations about the use of glucometers in glucose tolerance tests

**DOI:** 10.1515/almed-2024-0176

**Published:** 2024-11-25

**Authors:** Bernardo Alio Lavin-Gomez, Armando Raúl Guerra Ruíz

**Affiliations:** Hospital Universitario Marques de Valdecilla Ringgold Standard Institution – Analisis Clinicos, Santander, Cantabria, Spain

**Keywords:** glucose, measurements, POCT, enhanced glucometers

To the Editor,

We read with great interest the paper by Fabre-Estremera, B. et al. [1]. Congratulations for your article, which addresses a very relevant issue in the field of clinical biochemistry. The article provides a comparative study of POCT glucometers for professional use and measurement at the core laboratory to test for glucose tolerance in the diagnosis of prediabetes and diabetes. The paper also examines connectivity to the patient’s medical record and the associated cost reduction. Glucose test is a fundamental parameter in the diagnosis and management of patients with diabetes or glucose intolerance. Accurate glucose measurement is crucial in both in-hospital and out-of-hospital clinical activity. However, glucometers are often designed for self-monitoring rather than for professional use. Please, find below some critical considerations that may contribute to the debate about this issue.

1. Pre-analytical sample handling: We find it noteworthy that, to prevent glycolysis during overload tests, containers with glycolysis inhibitors were not used at all time points. Current guidelines recommend using rapidly effective glycolysis inhibitor tubes. If this is not feasible, the tube containing the sample should be immediately placed in an ice-water slurry and centrifuged within 30 min from sample collection [2]. Failure to comply with these recommendations may lead to underestimate actual blood glucose concentrations due to post-collection glycolysis. This negatively affects comparative analysis of glucometers.
2. Correlation between glucose measurements. The authors found statistically significant differences in fasting glucose concentrations, with glucometers showing higher values. Although these differences could be explained by the pre-analytical considerations mentioned in the section above and in the Discussion by the authors, the behavior of the samples on the regression analysis is remarkable. The regression plots and Passing-Bablok regressions in the Supplementary Material show a clear tendency of differences to increase significantly as glucose values increase in the core laboratory. This suggests a systematic bias at higher glucose values, with POCT yielding *lower* values as compared to the laboratory. Do you have any hypothesis that may explain this unexpected trend?
3. Last, but not least, several studies suggest that abnormal hematocrit values may interfere with glucose readings on glucometers [3], [4], [5]. In general, low hematocrit values tend to lead to falsely elevated glucose readings, whereas high hematocrit values induce low glucose readings. In pediatric patients, it is recommended to establish a different range for the hematocrit based on age and sex. Thus, limits should range from 34 to 54 %, which may lead to significant differences [6]. However, most glucometers consider a constant hematocrit of 43 %, as recommended by the IFFC [7]. The same guidelines recommend using a constant factor of 11 % (F=1.11) for converting glucose in whole blood to glucose in plasma, a factor that is generally used by manufacturers.

Table 1 shows the results (unpublished data) obtained for glycemia in heparinized plasma measured in the core laboratory (Atellica^®^Solution-CH; Siemens-Healthineers, Erlangen, Germany) using the enzymatic method (glucose hexokinase), as compared to glycemia in heparinized whole blood measured on a professional glucometer that does not adjust the result to the real hematocrit of the patient and another commercially-available glucometer that concomitantly measures the hematocrit and performs separate conversion for each individual sample. When the glucose value was not adjusted for the actual hematocrit, a marked tendency was observed in the glucometer to yield higher glucose values at lower hematocrit values and vice versa (maximum rise of 23 % and maximum reductions of 19 %, respectively) despite the use of a constant conversion factor. The two glucometers complied with ISO 15197:2013 recommendations (at least 95 % of values within ±15 mg/dL for glycemias <100 mg/dL, and ±15 % for glycemias ≥100 mg/dL).

**Table 1:** Glucose concentrations (mg/dL) in heparinized plasma in the Core Laboratory (method: glucose hexokinase, Atellica^®^ Solution-CH; Siemens-Healthineers) Vs. glucose concentrations in heparinized whole blood as measured on POCT glucometers.

Hematocrit, %	Glucose, mg/dL mean (CV%)		
(range)	Core lab	Gluc-A	Gluc-B
20–25	48 (2.1)	55 (1.9)	50 (2.2)
20–25	125 (1.2)	156 (1.3)	130 (1.2)
20–25	241 (1.5)	265 (1.8)	250 (1.7)
41–45	48 (2.1)	50 (1.9)	49 (2.2)
41–45	125 (1.2)	127 (1.3)	127 (1.2)
41–45	241 (1.5)	238 (1.8)	240 (1.7)
54–61	48 (2.1)	45 (1.9)	47 (2.2)
54–61	125 (1.2)	102 (1.3)	120 (1.2)
54–61	241 (1.5)	220 (1.8)	237 (1.7)

Gluc-A: glucometer not adjusting the result to the actual hematocrit – Freestyle Optium Neo (Abbott, Lake, IL, USA); electrochemical method, Glucose Dehydrogenase. Gluc-B: glucometer adjusting the result to the actual hematocrit – Nova StatStrip (Nova-Biomedical, Waltham, USA); electrochemical method, Glucose Oxidase. CV, analytical variability with respect to the mean value.

We agree with the authors that improvements in glucometers, both in analytical capabilities and in connectivity to electronic health records and full traceability, will increase their role in certain healthcare processes, reducing diagnostic and treatment delays, as well as analytical and clinical costs.
